# Prevalence of Sarcopenia and Its Effect on Postoperative Complications in Patients with Crohn's Disease

**DOI:** 10.1155/2021/3267201

**Published:** 2021-09-24

**Authors:** Chen Zhang, Dingye Yu, Liwen Hong, Tianyu Zhang, Hua Liu, Rong Fan, Lei Wang, Jie Zhong, Zhengting Wang

**Affiliations:** ^1^Department of Gastroenterology, Ruijin Hospital, Shanghai Jiaotong University School of Medicine, Shanghai, China; ^2^Department of Gastrointestinal Surgery, Ruijin Hospital, Shanghai Jiaotong University School of Medicine, Shanghai, China

## Abstract

**Background and Aims:**

Sarcopenia is a prognostic factor of outcomes for various diseases, but reports on sarcopenia in patients with Crohn's disease (CD) are few. We aim to determine the prevalence of sarcopenia and assess the role of sarcopenia in postoperative complications in patients with CD at a tertiary referral center.

**Methods:**

Patients who underwent intestinal surgery for CD from January 2013 to October 2019 were retrospectively enrolled. The L3 skeletal muscle mass index (SMI) was used to identify sarcopenia. Demographic data, preoperative laboratory data, surgical details, and hospital outcomes were recorded. The factors associated with postoperative complications were evaluated through univariate and multivariate analyses.

**Results:**

One hundred and twenty-four patients were enrolled. Thirty-four of them (27.4%), including 11 males, were diagnosed with sarcopenia. Compared with patients without sarcopenia, sarcopenic patients had a significantly lower BMI (*P* < 0.001); lower preoperative serum albumin (*P* = 0.006), prealbumin (*P* = 0.030), and hemoglobin levels (*P* < 0.001); longer hospital stay (34.4 ± 26.8 days vs. 22.8 ± 15.6 days, *P* = 0.003); and more occurrences of complications (41.2% vs. 23.3%, *P* = 0.049). The overall incidence of postoperative complications was 28.2%. Infection (51.4%) and intestinal fistula (22.9%) were the most common among such complications. Through the multivariate analysis, sarcopenia was identified as an independent risk factor for major postoperative complications (odds ratio = 3.974, 95%CI = 1.171–13.489, *P* = 0.027).

**Conclusion:**

Sarcopenia is common in patients with CD requiring bowel resection, and it significantly increases the risk of major postoperative complications.

## 1. Introduction

Crohn's disease (CD) is a nonspecific chronic inflammatory disease that affects any segment of the gastrointestinal tract and often causes extraintestinal complications [1–3]. Although the clinical drugs currently available for CD treatment are diverse, up to 80% of individuals with CD undergo at least one operation during their lifetime due to the complex complications of the disease, lack of response to medical treatment, and even malignant transformation in rare instances [4–6]. The incidence of surgical complications in patients with CD ranges from 20% to 40%; these complications include anastomotic leakage, wound rupture and infection, intra-abdominal septic complications, and short bowel syndrome [7]. A number of studies have been conducted to identify the risk factors for patients with CD undergoing surgery. Several risk factors, such as age, hypoalbuminemia, and anemia, are associated with postoperative outcomes [8–11]. However, the role of sarcopenia was not described well in these studies.

Sarcopenia, which is defined as a depletion in lean muscle mass accompanied with a loss of muscle strength, was first described in 1989 by Rosenberg. It is generally developed in aged patients or malnourished individuals with risk factors, such as chronic inflammation, oxidative stress, and hormonal changes [12–15]. Many studies have linked sarcopenia with poor postoperative outcomes in patients with colorectal cancer [16], pancreatic cancer [17], urological cancer [18], and hepatocellular carcinoma [19]. According to a systematic review, the incidence of sarcopenia is as high as 52% in patients with CD when anatomical criteria are considered without functional strength assessment [13, 20, 21]. However, the correlation between sarcopenia and postoperative complications remains unclear.

This study examined 124 patients with CD who underwent bowel resection. The objective is to assess (1) the prevalence of sarcopenia in patients with CD undergoing bowel resection, (2) evaluate the influence of sarcopenia as a risk factor for postoperative complications on these patients, and (3) compare the BMI, serum albumin level, prealbumin level, and other possible risk factors for postoperative complications of the patients with CD.

## 2. Methods

### 2.1. Study Design

The institutional ethics board approved this study. Informed consent was acquired from all patients. Patients who underwent CD-related bowel surgery from January 2013 to October 2019 in our hospital were retrospectively enrolled. CD-related bowel surgery is defined as surgery to cope with major complications, such as obstruction, leakage, and refractory abscess, in CD. The exclusion criteria were as follows: (1) computed tomography (CT) data not available 90 days before surgery or 30 days after surgery; (2) with severe comorbidity, important organ insufficiency, malignancy, or infection with HIV; (3) history of abdominal surgery; and (4) perianal surgery.

### 2.2. Data Collection

Demographic data included age, gender, marriage status, BMI, smoking history, and alcohol use. Normal BMI, overweight, obesity, and malnutrition were defined as 18.5–25 kg/m^2^, 25–30 kg/m^2^, >30 kg/m^2^, and <18.5 kg/m^2^, respectively. Clinical data included disease duration, Montreal classification, and preoperative medication. Disease activity was assessed using the Harvey–Bradshaw index (HBI). Laboratory test results, including serum albumin levels, prealbumin concentration, white blood cell (WBC) count, hemoglobin, and platelet count, were routinely recorded before surgery. Postoperative complications were registered mainly as skin and soft tissue infections, sepsis, venous thrombosis requiring treatment, anastomotic leak, or intra-abdominal abscess.

### 2.3. Skeletal Muscle Mass Index

**Figure 1:**
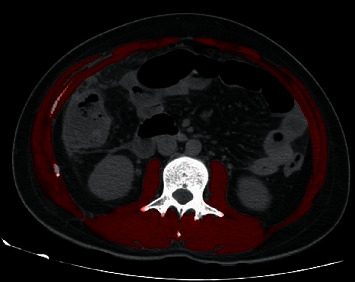
Evaluation of the skeletal muscle mass using a third lumbar computed tomography scan slice. Red: skeletal muscle (including the psoas, paraspinal, and abdominal wall muscles).

The skeletal muscle mass index (SMI) of each patient was rated based on the skeletal muscle mass measured through CT of the abdomen and pelvis. CT scans within three months prior to surgery or in the first month after surgery were selected, and preoperative scans were preferentially used. The total muscle cross-sectional area (cm^2^) at L3 vertebra was utilized for the segmentation of skeletal muscle (including the psoas, paraspinal, and abdominal wall muscles). The threshold range for skeletal muscle was from −30 to +150 Hounsfield units (HU) in accordance with reports (Figure 1) [22]. Sarcopenia was identified based on SMI (cm^2^/m^2^), which is the ratio of the skeletal muscle area (cm^2^) to the height squared (m^2^). In accordance with a previous work, sarcopenia was identified once a patient fulfilled one of the following criteria: (1) SMI < 41 cm^2^/m^2^ in women, (2) <43 cm^2^/m^2^ in men with BMI < 25 kg/m^2^, and (3) <53 cm^2^/m^2^ in men with BMI ≥ 25 kg/m^2^ [23].

### 2.4. Statistical Analyses

All statistical analyses were performed using SPSS (version 23.0; Inc, Chicago, IL, United States). The mean value and SD were calculated for quantitative and qualitative variables. Data between groups were compared using Student's *t*-test for normally distributed values, and categorical data were compared using *χ*^2^ or Fisher's exact test, as appropriate. Univariate and multivariate logistic regression analyses were performed to identify the independent predictors of postoperative complications. The Kaplan–Meier curve was applied to estimate the impact of sarcopenia on the length of hospital stay. Linear regression was performed to analyze the linear relationship between SMI and BMI, albumin, and prealbumin level. *P* < 0.05 was considered statistically significant.

## 3. Results

### 3.1. Demographic and Clinical Characteristics of CD Patients with or without Sarcopenia

A total of 124 patients who underwent CD-related bowel surgery in our hospital from January 2013 to October 2019 were included in this work. The baseline characteristics of the patients are shown in Table 1. The prevalence of sarcopenia was 27.4% (34/124), in which 32.4% (11/34) was observed in men. This percentage is significantly lower than that for the no-sarcopenia group (*P* < 0.001). Smoking was more prevalent in patients without sarcopenia (*P* = 0.035) than in patients with sarcopenia. Sarcopenic patients had a significantly lower BMI (16.75 ± 2.59 vs. 19.49 ± 3.03, *P* < 0.001), lower serum albumin levels (29.2 ± 6.1 g/L vs. 33.0 ± 8.0 g/L, *P* = 0.006), lower prealbumin levels (141.8 ± 70.2 vs. 175.0 ± 84.9 mg/L, *P* = 0.030), and lower hemoglobin levels (98.3 ± 18.8 vs. 115.1 ± 24.4 g/L, *P* < 0.001) than those without sarcopenia. With regard to disease behavior, the sarcopenic patients had strictures and low B1 and B3 (*P* = 0.026). No significant differences were observed between the two groups in terms of age, disease duration, disease location, perianal disease, preoperative medication, and HBI score (Table 1).

### 3.2. Surgical Details of CD Patients with or without Sarcopenia

The surgical details of CD patients with or without sarcopenia are listed in Table 2. Twelve percent of the patients had emergency surgery. No significant difference in indication for surgery was found between the groups with and without sarcopenia. The main indications for surgery were strictures (46.0%) and penetrating complications (29.0%). Laparoscopic operations were performed in 26.6% of the cases. Colostomy was performed in 40.3% of the patients, with no significant difference between the two groups.

### 3.3. Analysis of Postoperative Outcomes and Complications in CD Patients with or without Sarcopenia

**Figure 2:**
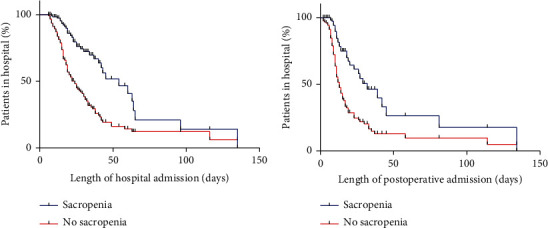
Length of overall hospital stay and postoperative hospital stay dependent on with or without sarcopenia. (a) Length of overall hospital stay (34.41 ± 26.83 vs. 22.79 ± 15.59 days, *P* = 0.003). (b) Length of postoperative hospital stay (22.47 ± 24.73 vs. 14.08 ± 13.72 days, *P* = 0.018).

The postoperative outcomes and complications are listed in Table 3. The length of hospital admission was 25.98 ± 19.90 days (16.38 ± 17.73 days of postoperative stay). A significant difference was observed in the length of hospital stay and postoperative stay between patients with sarcopenia and those without (34.41 ± 26.83 vs. 22.79 ± 15.59 days, *P* = 0.003; 22.47 ± 24.73 vs. 14.08 ± 13.72, *P* = 0.018) (Figure 2). Sixty-three patients required PN therapy postoperatively. In total, 35 complications were reported (28.2%) (Table 3). The sarcopenic CD patients had much more complications (41.2% vs. 23.3%, *P* = 0.049) than the nonsarcopenic CD patients. Of the 124 patients, 12 (9.7%) required hospital readmission within 30 days of surgery, among which 3 patients required ICU admission and 2 patients needed reoperations. No significant difference was found in terms of hospital readmission, reoperation, ICU admission, and death between sarcopenic and nonsarcopenic groups.

### 3.4. Univariate and Multivariate Analysis for Postoperative Complications

Statistical analyses were performed to identify the potential risk factors for the occurrence of postoperative complications. In the univariate analysis, low BMI (*P* = 0.049), low HBI (*P* = 0.027), low preoperative levels of serum albumin (*P* = 0.001), low prealbumin levels (*P* = 0.009), emergency surgery (*P* = 0.002), and colostomy and sarcopenia (*P* = 0.026) were identified as factors related to postoperative complications (Table 4). In the multivariate regression analysis, sarcopenia (OR: 3.974; 95% CI: 1.171–13.489; *P* = 0.027) and male gender (OR: 4.080; 95% CI: 1.205–13.814; *P* = 0.024) were identified as independent risk factors associated with postoperative complications (Table 4).

### 3.5. Factors That Affect SMI in Patients with CD

Multiple linear regression analyses were performed to investigate possible associations between SMI and disease duration, BMI, body weight, serum albumin levels, and prealbumin levels. The values for body weight and serum albumin levels were significantly correlated with SMI (*R*^2^ = 0.52, Table 5). The higher the albumin level was, the higher SMI was and the lower the prevalence of sarcopenia was. Body weight exerted more influence on SMI than albumin level. The linear regression equation is shown in Table 5.

## 4. Discussion

Many studies indicate that CD may cause skeletal muscle loss or sarcopenia [22, 24, 25]. However, the prevalence of sarcopenia among patients with CD varies significantly in relevant studies. Schneider et al. discovered that the prevalence of sarcopenia is high at 60% among remission phase CD patients through measurements obtained via dual-energy X-ray absorptiometry (DEXA) [21]. Zhang et al. obtained a prevalence rate of 61.4% in 114 CD patients in China through the measurement of the skeletal muscle area via abdominal CT with SMI of less than 55 cm^2^/m^2^ in male and SMI of less than 39 cm^2^/m^2^ in female patients [20]. A US study in Iowa calculated the total psoas index via CT among IBD patients and derived a 24.7% prevalence rate of sarcopenia [25].

In this study, we found a sarcopenia prevalence of 27.4% based on the threshold set by Martin et al., who reported an SMI of less than 41 cm^2^/m^2^ in females and an SMI of less than 43 cm^2^/m^2^ (BMI < 25 kg/m^2^) or SMI of less than 53 cm^2^/m^2^ (BMI ≥ 25 kg/m^2^) in males. The prevalence in this study is within the range in other literature but relatively low [20–22, 26, 27]. On one hand, this is because the measurement methods in various studies differed either due to DEXA being affected by fat tissue and body water or the different SMI thresholds used. On the other hand, the majority of the patients in this study underwent elective surgery and have thus been receiving total or partial enteral nutrition support, which might lead to the reduced prevalence of sarcopenia. We also discovered that 14.7% of sarcopenia patients in the study were of normal weight or overweight. Therefore, they are not identified as malnourished under the traditional body mass index (BMI) method. The diagnosis of sarcopenia is important to patients' nutrition evaluation and treatment, and even normal-weight and overweight patients deserve special attention.

A few studies have indicated that the incidence of sarcopenia can cause multiple adverse events, such as osteoporosis with pathologic fracture, repeated hospitalization, mobility difficulty, and reduction in the quality of life [11–13, 25]. Chen et al. diagnosed 11.8% of 313 gastric cancer patients with postlaparoscopic gastrectomy as sarcopenic [28]. Sarcopenia significantly increased the postoperative complications, hospital stay days, and total financial costs of the patients with sarcopenia compared with the patients without it [26, 29].

Reports on postoperative complications among CD patients are scarce. Therefore, the 124 postoperative patients were studied using logistic single-factor regression and multiple-factor regression, which revealed that only sarcopenia (OR = 3.974, *P* = 0.027) and male gender (OR = 4.080, *P* = 0.024) can be considered independent risk factors for postoperative complications. SMI can reflect patients' nutrition status and predict the incidence of complications more accurately compared with conventional nutrition indicators, such as BMI, prealbumin level, and albumin level [9, 11, 30]. Therefore, improving patients' perioperative nutrition status is important in reducing the occurrence of sarcopenia and postoperative complications.

Research has shown that elevation of serum cytokines, such as tumor necrosis factor-*α* (TNF-*α*) and interleukin 6, reduces insulin-like growth factor-1 (IGF-1) in the serum and muscle of CD patients. This reduction, in turn, results in growth hormone resistance in the liver and skeletal muscle, leading to a downward moderation of the mTOR pathway into activation of the ubiquitin ligand and expression of proteolytic enzyme, which cause skeletal muscle mass reduction and impairment of muscle contraction [13, 14]. Current interventions for sarcopenia include exercise therapy, nutrition therapy, and medication. Most experts recommend that CD patients undergo exercise therapy for 6 weeks to 3 months to improve the patients' general condition, increase the oxygenation index, and correct malnutrition [3, 10]. The guidelines of the European Society for Clinical Nutrition and Metabolism recommend that active (adult) IBD patients increase their protein intake to 1.2–1.5 g/kg/d (with 50% high-quality protein) higher than that recommended for the general population to increase the skeletal muscle cell volume, inhibit proteolysis, and reverse muscle mass reduction and functionality decline [31]. Medication for sarcopenia is still in the exploration phase, and no specific drug has been invented yet. However, Subramaniam et al. proved that a TNF-*α* inhibitor, infliximab, can inhibit the activation of NF-*κ*B, reduce proteolysis and sarcolysis, and accelerate muscle formation to reverse sarcopenia [32]. Thus, gastroenterologists, surgeons, radiologists, and nutritionists should work closely together to determine an early intervention plan and a proper operation timing to reduce the incidence of postoperative complications.

However, the current study has several potential limitations. First, selection bias may exist in this retrospective work. Second, the lack of consensus regarding the adoption of an SMI threshold remains unaddressed. This study used the widely applied CT measurement method proposed by Martin et al., so it cannot be compared directly with other studies. Third, this retrospective study's diagnosis of sarcopenia consisted of skeletal muscle mass reduction and muscle strength decline, which requires the measurement of grip strength and walking pace. These measurements could be performed in any further prospective study to achieve an accurate diagnosis of sarcopenia. Despite these limitations, this study revealed that sarcopenia can be used as an independent risk factor to predict the incidence of complications in patients with CD.

## 5. Conclusion

Sarcopenia is common in patients with CD and is unlikely to be recognized by a routine clinical assessment using BMI alone. Detection of sarcopenia is important; it can serve as a prognostic factor for the prediction of postoperative complications in patients with CD undergoing bowel resections. Therefore, the use of the SMI index together with a routine assessment of nutritional status should be a cornerstone in clinical practice.

## Figures and Tables

**Table 1:** Demographic and clinical characteristics in CD patients with or without sarcopenia.

	All patients (*n* = 124)	Sarcopenia (*n* = 34)	Nonsarcopenia (*n* = 90)	*P* value
Age, years	37.06 ± 13.08	37.03 ± 16.55	37.08 ± 11.61	0.985
Gender				*<0.001*
Male	74 (59.7)	11 (32.4)	63 (70.0)	
Smoking	12 (9.7)	0 (0)	12 (13.3)	*0.035*
Alcohol	7 (5.6)	0 (0)	7 (7.8)	0.188
BMI	18.74 ± 3.15	16.75 ± 2.59	19.49 ± 3.03	*<0.001*
BMI categories				*<0.001*
Underweight	59 (47.6)	29 (85.3)	30 (33.3)	
Normal	57 (46.0)	4 (11.8)	53 (58.9)	
Overweight	8 (6.5)	1 (2.9)	7 (7.8)	
Skeletal muscle index (cm^2^/m^2^)	53.89 ± 15.13	37.98 ± 3.94	59.90 ± 13.32	*<0.001*
Disease duration (months)	55.81 ± 51.67	54.49 ± 54.45	56.31 ± 50.89	0.867
Age of onset				0.492
≤16 (A1)	9 (7.3)	4 (11.8)	5 (5.6)	
17-40 (A2)	81 (65.3)	21 (61.8)	60 (66.7)	
>40 (A3)	34 (27.4)	9 (26.5)	25 (27.8)	
Disease location				0.063
Ileum (L1)	71 (57.3)	16 (47.1)	55 (61.1)	
Colon (L2)	18 (14.5)	9 (26.5)	9 (10.0)	
Ileocolon (L3)	35 (28.2)	9 (26.5)	26 (28.9)	
Disease behavior				*0.026*
Nonstricturing, nonpenetrating (B1)	22 (17.7)	4 (11.8)	18 (20.0)	
Stricturing (B2)	67 (54.0)	25 (73.5)	42 (46.7)	
Penetrating (B3)	35 (28.2)	5 (14.7)	30 (33.3)	
Perianal disease	34 (27.4)	13 (38.2)	21 (23.3)	0.097
HBI score	6.39 ± 2.81	6.94 ± 2.33	6.18 ± 2.96	0.179
Preoperative therapy				0.712
No therapy	22 (17.7)	8 (23.5)	14 (15.6)	
Steroid	20 (16.1)	6 (17.6)	14 (15.6)	
5ASA	24 (19.4)	8 (23.5)	16 (17.8)	
AZA	36 (29.0)	8 (23.5)	28 (31.1)	
MTX	4 (3.2)	1 (2.9)	3 (3.3)	
IFX	18 (14.5)	3 (8.8)	15 (16.7)	
Hemoglobin (g/L)	110.5 ± 24.1	98.3 ± 18.8	115.1 ± 24.4	*<0.001*
White cell count (cells ×109/L)	6.94 ± 3.97	6.84 ± 4.37	6.98 ± 3.83	0.872
Platelet	265 ± 119	293 ± 130	254 ± 114	0.128
Prealbumin (g/L)	165.9 ± 82.2	141.8 ± 70.2	175.0 ± 84.9	*0.030*
Albumin (g/L)	31.9 ± 7.7	29.2 ± 6.1	33.0 ± 8.0	*0.006*

BMI: body mass index; HBI: Harvey Bradshaw Index; 5-ASA: 5-aminosalicylate; AZA: Azathioprine; MTX: Methotrexate; IFX: Infliximab. *P* values < 0.05 were highlighted in italic.

**Table 2:** Surgical details in CD patients with or without sarcopenia.

	All patients (*n* = 124)	Sarcopenia (*n* = 34)	Nonsarcopenia (*n* = 90)	*P* value
Emergency surgery	16 (12.9)	2 (5.9)	14 (15.6)	0.152
Indication for surgery				0.365
Bowel obstruction	57 (46.0)	18 (52.9)	39 (43.3)	
Fistula	14 (11.3)	5 (14.7)	9 (10.0)	
Medically refractory disease	17 (13.7)	2 (5.9)	15 (16.7)	
Perforation	36 (29.0)	9 (26.5)	27 (30.0)	
Type of surgery				0.374
Open	91 (73.4)	23 (67.6)	68 (75.6)	
Laparoscopic	33 (26.6)	11 (32.4)	22 (24.4)	
Colostomy	50 (40.3)	16 (47.1)	34 (37.8)	0.347

**Table 3:** Analysis of postoperative outcomes and complications in CD patients with or without sarcopenia.

	All patients (*n* = 124)	Sarcopenia (*n* = 34)	Nonsarcopenia (*n* = 90)	*P* value
Length of hospital admission (overall)	25.98 ± 19.90	34.41 ± 26.83	22.79 ± 15.59	*0.003*
Length of hospital admission (postoperation)	16.38 ± 17.73	22.47 ± 24.73	14.08 ± 13.72	*0.018*
Parenteral nutrition	63 (50.8)	20 (58.8)	43 (47.8)	0.272
Complications	35 (28.2)	14 (41.2)	21 (23.3)	*0.049*
Skin or soft tissue infection	18 (14.5)	6 (17.6)	12 (13.3)	
Major intra-abdominal leak	8 (6.5)	4 (11.8)	4 (4.4)	
Postoperative sepsis	4 (3.2)	2 (5.9)	2 (2.2)	
Postoperative thrombosis	2 (1.6)	1 (2.9)	1 (1.1)	
Organ space infection	3 (2.4)	1 (2.9)	2 (2.2)	
Hospital readmission within 30 days	12 (9.7)	4 (11.8)	8 (8.9)	0.629
Reoperation	2 (1.6)	2 (5.9)	0 (0.0)	0.074
ICU admission	3 (2.4)	1 (2.9)	2 (2.2)	0.621
Death	1 (0.8)	1 (2.9)	0 (0.0)	0.274

*P* values < 0.05 were highlighted in italic.

**Table 4:** Univariate and multivariate regression analysis for risk factors of postoperative complication.

	Univariate analysis			Multivariate analysis		
Variable	OR	95% CI	*P* value	OR	95% CI	*P* value
Male	2.041	0.878-4.746	0.098	4.080	1.205-13.814	*0.024*
Smoking	2.089	0.434-10.055	0.358			
Alcohol	2.458	0.285-21.191	0.413			
BMI			*0.049*			0.147
Underweight	4.308	0.499-37.207	0.184	6.353	0.444-90.812	0.173
Normal	1.628	0.179-14.771	0.625	2.501	0.165-37.947	0.509
Overweight	1			1		
Sarcopenia	2.587	1.124-5.959	*0.026*	3.974	1.171-13.489	*0.027*
Duration (months)	0.996	0.988-1.004	0.313			
Age of onset			0.493			
<16 years (A1)	1					
16-40 years (A2)	1.677	0.496-5.665	0.405			
>40 years (A3)	1.037	0.265-4.052	0.958			
Disease location			0.493			
Ileum (L1)	1					
Colon (L2)	0.964	0.247-3.768	0.958			
Ileocolon (L3)	1.617	0.636-4.109	0.312			
Disease behavior			0.371			
Nonstricturing, nonpenetrating (B1)	1					
Stricturing (B2)	0.635	0.199-2.029	0.443			
Penetrating (B3)	0.531	0.219-1.288	0.161			
Perianal disease	1.742	0.678-4.475	0.249			
HBI score			*0.027*	1.167	0.911-1.495	0.381
Remission (<5)	1					
Mild active (5-8)	3.103	1.240-7.761	*0.016*	2.485	0.674-9.160	0.172
Sever active (>8)	4.583	1.124-18.693	*0.034*	2.591	0.376-17.843	0.334
Hemoglobin	0.988	0.972-1.004	0.141	1.000	0.974-1.028	0.986
White cell count	1.098	0.998-1.208	0.056	0.947	0.826-1.085	0.431
Platelet	1.001	0.998-1.004	0.552			
Prealbumin (g/L)	0.992	0.986-0.998	*0.009*	0.994	0.982-1.007	0.362
Albumin (g/L)	0.911	0.860-0.965	*0.001*	1.014	0.993-1.152	0.830
Emergency surgery	5.533	1.830-16.731	*0.002*	4.225	0.730-24.459	0.108
Indication for surgery			*0.049*			0.687
Stricture	1			1		
Fistula	2.323	0.649-8.321	0.195	1.383	0.251-7.625	0.710
Medically refractory disease	0.896	0.219-3.670	0.879	3.221	0.457-22.695	0.240
Perforation	3.345	1.320-8.479	*0.011*	1.258	0.382-4.140	0.706
Type of surgery	2.754	0.967-7.848	0.058	1.393	1.345-5.633	0.642
Colostomy	4.401	1.916-10.108	*<0.001*	1.965	0.665-5.805	0.222

BMI: body mass index; HBI: Harvey Bradshaw Index; 5-ASA: 5-aminosalicylate; AZA: Azathioprine; MTX: Methotrexate; IFX: Infliximab. *P* values < 0.05 were highlighted in italic.

**Table 5:** Factors affecting skeletal muscle mass index in patients with CD.

Variable	*β*	95% CI	*P* value
Body weight	0.939	0.758-1.119	*<0.001*
Serum albumin level	0.355	0.103-1.606	*0.006*

Linear regression equation *Y* = −7.310 + 0.939∗body weight + 0.355∗albumin*R*^2^ = 0.520.

## Data Availability

The datasets used or analyzed during the current study are available from the corresponding author on reasonable request.
